# Cerebellar Transcranial Direct Current Stimulation Improves Reactive Response Inhibition in Healthy Volunteers

**DOI:** 10.1007/s12311-019-01047-z

**Published:** 2019-06-08

**Authors:** Syanah C. Wynn, Josi M. A. Driessen, Jeffrey C. Glennon, Inti A. Brazil, Dennis J. L. G. Schutter

**Affiliations:** 1 Donders Institute for Brain, Cognition and Behaviour, Radboud University, P.O. Box 9104, 6500 HE Nijmegen, The Netherlands; 2 Donders Institute for Brain, Cognition and Behaviour, Radboud University Medical Centre, 6525 GA Nijmegen, The Netherlands

**Keywords:** Cerebellum, Executive functions, Cognition, Inhibition, Transcranial direct current stimulation

## Abstract

Involvement of the cerebellum to non-motor related aspects of behavior is becoming increasingly clear. The aim of this study was to investigate the role of the cerebellum in reactive and proactive behavioral control and interference. In a double-blind controlled within-subject design, 26 healthy volunteers underwent real and sham cerebellar transcranial direct current stimulation (tDCS) while performing a go/no-go task and a delay discounting task. Results showed that the number of go/no-go commission errors was significantly lower during real as compared with sham cerebellar tDCS. No effects of tDCS were observed on delay discounting. Our findings provide further behavioral support for the involvement of the cerebellum in fast neural processes associated with response inhibition.

## Introduction

The classical view that the cerebellum is primarily involved in motor-related functions is being challenged by a rapidly growing body of evidence in support of cerebellar involvement in cognitive and affective processes. Neuropsychological studies have, for instance, demonstrated that cerebellar damage can cause impulsivity, emotion dysregulation, and problems in behavioral inhibition [1–5]. This pattern of non-motor-related symptoms is part of the cerebellar cognitive affective (Schmahmann’s) syndrome and is attributed to damage of the posterolateral parts of the cerebellum [4, 6, 7]. More recent functional neuroimaging studies have confirmed the role of the cerebellum in the regulation of both motor, affective, and cognitive processes [8–13]. Furthermore, results of a recent fMRI study showed that neural activities in the cerebellum, cingulate cortex, and cortical association areas are linked to interference control when there is competing information [14]. Even though the neural mechanisms remain elusive, research data point towards the involvement of cerebello-cortical and cerebello-cortical-subcortical loops in behavioral control and inhibition. This is substantiated by findings showing activation of the cerebellar-thalamo-frontal and cerebellar-thalamo-striatal pathways when a prepotent motor response needs to be inhibited [15, 16]. In addition, cerebellar activity and functional cerebello-frontal cortical connectivity are inversely related to the preference for a small immediate reward over a larger delayed reward [17–20]. Collectively, these studies suggest that the cerebellum is involved in several aspects of behavioral and interference control that vary from rapid inhibition of simple motor responses to slower, more complex executive control. Reactive inhibition refers to the suppression of fast, simple motor responses, for instance, when occasionally a prepotent response needs to be withheld. In contrast, proactive inhibition deals with slower, complex, anticipatory executive control, for example when choosing between a smaller immediate reward and a larger delayed reward.

Cerebellar transcranial direct current stimulation (tDCS) is increasingly used in both research and clinical settings to modulate motor, cognitive, and affective functions [21, 22]. At this point, the precise mechanisms of action underlying the neuronal effect of cerebellar tDCS are still unknown [22]. As a consequence, it is difficult to predict the direction of the behavioral effect in healthy volunteers (see also [3]). Following the simplified assumption that cathodal tDCS reduces neural excitability [21, 23], the current double-blind sham-controlled crossover study applied cathodal cerebellar tDCS in order to reduce response inhibition in healthy volunteers. More specifically, we hypothesized a reduction in fast motor inhibition and a decline in anticipatory executive control during cathodal cerebellar tDCS. However, results from a recent meta-analytical study indicate that the polarity of cerebellar tDCS is not predictive for the direction of performance change [3], and thus, cathodal tDCS to the cerebellum may also enhance reactive and proactive inhibition.

## Material and Methods

### Participants

Twenty-six healthy individuals (19 women) aged between 18 and 30 years (*M* = 23.48, *SD* = 2.55) participated in this study. All had normal or corrected-to-normal vision, were native Dutch speakers, non-smokers, and right handed. Main exclusion criteria were as follows: metal in the cranium, epilepsy or a family history of epilepsy, history of other neurological conditions or psychiatric disease, heart disease, use of psychoactive medication or substances, and pregnancy. All volunteers received 10 euros per hour for participation. The study was approved by the medical ethics committee of the Radboud University Medical Centre, Nijmegen, the Netherlands, and was carried out in accordance with the standards of the declaration of Helsinki.

### Reactive Response Inhibition: Go/No-Go Task

A commonly used version of the go/no-go task was utilized to measure reactive response inhibition of prepotent motor responses [24]. Stimulus presentation and recording of responses were attained using E-Prime (v2.0; Psychology Software Tools, Pittsburgh, PA). The task consists of 225 trials in which a digit, ranging from 1 to 9, was presented at the center of the screen for a duration of 250 ms. Digits were presented in quasi-random order with an inter-trial interval of 850 ms. Participants were instructed to push the spacebar each time a digit other than “3” appeared on the screen (go trials). In 11.1% of all trials, a “3” appeared on the screen and the participants were instructed to withhold their response (no-go trials). The main task was preceded by a practice session of 20 trials. A commission error was defined by a key press in the case of a no-go trial, while an omission error was defined by the absence of a key press in go trials. The amount of commission errors was the main outcome measure of the task. In general, go/no-go tasks are well-validated measures of fast, simple motor inhibition [25, 26], with a positive correlation between motor inhibition and the number of commission errors [27, 28].

### Proactive Response Inhibition: Delay Discounting Task

A delay discounting task was administered to measure proactive inhibition, which requires executive control. Again, stimulus presentation and recording of responses were attained using E-Prime (v2.0; Psychology Software Tools, Pittsburgh, PA). The task consisted of 30 hypothetical trials in which participants chose between a relatively small monetary reward that they would receive the same day (immediate reward; IR) and a reward of 10 euros that they would receive after a certain delay (delayed reward; DR). To increase ecological validity and participant’s motivation, one of the trials was randomly selected and paid out as a bonus to the participant, in addition to the hourly monetary compensation. The amount of IR and the duration of the delay (2, 14, 30, 180, and 365 days) varied between trials. For each of the five different delays, participants were presented with six consecutive choices. On each delay, the first choice started with an IR of 5 euros. For the following choices, the IR depended on the previous choice of the participant [29]. As a result of choosing IR on the first trial, the amount of IR was decreased by half of the difference between the IR and DR on the next choice, whereas, as after choosing DR on the first trial, the amount was increased by half of the difference between the IR and DR on the next choice. For subsequent trials, as a result of choosing IR, the amount of IR was decreased by half of the previous adjustment, whereas, after choosing DR, the amount was increased by half of the previous adjustment. For each delay, the value of the IR after the sixth trial was used to estimate the subjective value of the DR, i.e., the indifference point. The subjective values were used to define the area under the curve (AUC), which was calculated according to the procedure in [30]. The first step of this procedure is the normalization of the delay and subjective values, by expressing the delay as a proportion of the maximal delay, and expressing the subjective value as a proportion of the DR. These normalized values were respectively used as *x* and *y* coordinates. When imaginary vertical lines are drawn from each data point to the *x* axis, the graph can be divided into trapezoids. The sum of the area of all these trapezoids was used as the AUC. The AUC is positively related to inhibition, where AUC ranges from 0 to 1. This method is commonly used to quantify delay discounting (e.g., [31–35]).

### Cerebellar Transcranial Direct Current Stimulation

A cathodal electrode was placed over the medial cerebellum, 2 cm below the inion with the electrode’s lateral borders 1 cm medially to the mastoid apophysis (size 35 cm^2^; current density 0.05 mA/cm^2^). The anodal electrode was placed over the right deltoid muscle [size 25cm^2^; current density 0.08 mA/cm^2^; 21]. The duration of the stimulation was 30 min, including a ramp-up and ramp-down period of 15 s in which the intensity gradually increased from 0 to 2 mA, or vice versa. During sham cerebellar tDCS, the ramp-up period was followed by 30 s of real stimulation after which the intensity was ramped down to 0 mA. Electrode impedance was kept below 10 kΩ throughout the experiment.

### Procedure

The current sham-controlled double-blind within-subject study design consisted of two test sessions which took place on two separate days, exactly 1 week apart. Prior to the experiment, participants received written and oral information about the study, after which they filled in the consent and screening forms. Thereafter, participants received either sham or active cerebellar tDCS while performing both the go/no-go and delayed discounting tasks. Stimulation conditions were randomized and counterbalanced across participants. At the end of each session, participants filled out an evaluation form and a checklist to validate the blinding procedure. After finishing the second test session, participants were debriefed and received monetary compensation.

### Data Analyses

Data were analyzed using MATLAB 2015b (Mathworks, USA) and SPSS version 25 (Armonk, NY). Before statistical tests were performed on the go/no-go task, trials with reaction times greater than three standard deviations from the mean were excluded. On average trials, 1.63% of the trials (*M* = 3.66, *SD* = 2.05) were excluded per participant. A repeated measure general linear model (GLM) was used to test for statistically significant within-subject effects of cerebellar tDCS on go/no-go commission errors and delay discounting AUC. The alpha level of significance was set to .05 (two tailed).

## Results

Stimulation was well tolerated, and no adverse events occurred. One of the participants did not adhere to the instructions of the go/no-go task and was therefore excluded from the analyses pertaining to this task. The blinding procedure was successful as participants were not able to distinguish real from sham tDCS (*χ*^2^(1) = 0.62, *p* = .43).

The GLM showed a significant main effect of tDCS condition on go/no-go commission errors (*F*(1,24) = 5.37, *p* = .03, *ηp*^2^ = .18). There were significantly fewer go/no-go commission errors observed during real (*M* = 11.56, *SD* = 4.19) as compared with the sham tDCS (*M* = 13.16, *SD* = 4.62; see Table 1). The AUCs did not differ significantly between real (*M* = 0.61, *SD* = 0.24) and sham (*M* = 0.59, *SD* = 0.26) stimulation (*F*(1,24) = 0.23, *p* = .63, *ηp*^2^ = .01).

**Table 1:** Go/no-go performance during real and sham cerebellar cathodal tDCS

	Real		Sham	
	Mean	SD	Mean	SD
Correct responses	193.08	8.45	193.04	7.53
Commission errors	11.56	4.19	13.16	4.62
Omission errors	16.48	6.81	15.36	5.57
RT correct Go	243.73	29.66	250.06	25.06
RT commission errors	231.12	42.54	232.94	38.31

## Discussion

The aim of the present study was to investigate the role of the cerebellum in proactive and reactive inhibition. Our results show that cathodal tDCS over the medial cerebellum, as compared with sham tDCS, reduced the percentage of commission errors during the go/no-go task, while performance on the delay discounting task was not affected. Since imaging studies reported cerebellar involvement in networks implicated in reactive inhibition [15, 16] and proactive inhibition [17–20], the latter finding is unanticipated. A possible explanation is that proactive inhibition, as currently measured with the delay discounting task, relies more on cortical regions, such as the dorsolateral prefrontal cortex which is involved in executive functions [36, 37]. The effect of cerebellar cathodal tDCS on the go/no-go task concurs with previous demonstrations of an association between reactive motor response inhibition and the cerebellar-striatal-cortical pathways [15, 16]. In particular, an inverse correlation between response inhibition and cerebellar activity during a serial response reaction time task has been reported [38]. This adds to the growing body of evidence supporting the contribution of the cerebellum to inhibitory control processes.

It should be noted that, at first glance, our findings are at odds with the general idea that cathodal tDCS interferes with cerebellar processes, having a negative effect on performance [21, 23]. However, various explanations can be put forward to account for the observed improvement in reactive response inhibition in our study. On the cellular level, an externally applied direct current polarizes Purkinje cells (PC) along the somato-dendritic axis in a linear fashion [39], and can be used to either depolarize (anodal tDCS) or hyperpolarize nerve cells (cathodal tDCS). The angle between the applied electric field and the somatic-dendritic axis of the PC is a critical factor in the magnitude and direction of polarization. Due to the complexity of gyral folding of the cerebellar cortex, tDCS can cause different polarization profiles at different sites [40]. Given the relatively broad electric field of tDCS, it is conceivable that parts of the cerebellum in fact show depolarization to cathodal tDCS. If these parts indeed play a significant role in task execution, then cathodal tDCS can facilitate processes and enhance performance. An alternative explanation for the findings is the modulation of GABA-ergic interneurons in the outer surface (molecular) layer of the cerebellar cortex. The molecular layer contains stellate and Basket cells that inhibit PC, and lowering GABA release by hyperpolarizing these interneurons through cathodal tDCS [41] may affect PC activity and cause a transient gain in the cerebellar output channels [42]. It should be noted that, similar to the parallel fibers in this layer, the horizontal organization of these neurons makes them less susceptible to tDCS. Then again, the orientation due to gyral folding differs across the cerebellum and, similar to the previous explanation, leaves open the possibility for neuromodulatory effects of tDCS. Another alternative may be through transsynaptic activation of extracerebellar areas following the polarization of PC. Cathodal tDCS may hyperpolarize PC and downregulate its inhibitory control over the deep cerebellar nuclei (DCN). The reduction in inhibitory PC activity causes excitation of the DCN and connected distal regions including the primary motor cortex. Additionally, the excitatory projections from the DCN to the reticular formation in the brainstem could increase general arousal in the central nervous system and boost performance [43].

The reason as to why we only found effects on reactive response inhibition may be related to task differences and the associated brain regions to perform the task. For example, without extensive practice, participants typically show an improvement in behavioral performance during the course of a task [44]. Purkinje cell simple spike suppression is widely regarded as part of the cerebellar mechanism underlying learning [45]. Indeed, simultaneous activation of parallel fibers (pf) and PC causes long-term depression (LTD) at the pf-PC synapse and has been found to facilitate learning and memory formation [46]. This explanation implies that the direction of the extracellular current flow is of less critical importance, which leads to the hypothesis that anodal tDCS should have a similar behavioral effect. Evidence for paradoxical facilitation of functions following confined brain lesions and non-invasive brain stimulation in healthy volunteers has been reported [47]. Furthermore, acquired cerebellar damage can hinder the initiation of response inhibition [48] with the most pronounced effects being observed in patients with lesions involving the DCN. These patients experienced difficulties during an infrequent and unpredictable event and were more prone to errors. This study not only further supports the importance of the DCN for understanding cerebellar functions but also leaves open the possibility that hyperpolarizing the cerebellar cortex may cause a shift to the DCN. This shift can be explained by reduced afferent inhibitory input and promotion of processes that do not rely on PC.

Even though neuropsychological studies have shown that cerebellar damage can affect reward-punishment sensitivity, impulsivity, and planning, cerebellar tDCS did not influence proactive inhibition in the current study. In retrospect, task properties may have been suboptimal in detecting improvements in healthy volunteers due to ceiling effects. While this argument also holds for reductions in proactive inhibition, another hypothetical explanation for the null effect may be related to successful compensation. Proactive inhibition is a complex cognitive phenomenon that involves many subcortical and cortical regions (Schüller et al. [49]). It is therefore conceivable that cerebellar tDCS triggered a compensatory process within these functionally connected regions. Evidence for this hypothesis comes from a recent study that found compensatory cortical activity in response to cerebellar tDCS during performance monitoring (Schutter et al., under review).

The current study applied cathodal cerebellar tDCS on healthy participants in order to investigate the role of the cerebellum in reactive and proactive response inhibition. Taking into account the inconsistent evidence regarding the polarity-dependent effects of cerebellar tDCS, the inclusion of an anodal condition would have strengthened the design. The current findings provide first evidence of cerebellar involvement in reactive response inhibition. Future studies including both cathodal and anodal tDCS conditions could provide further insight into polarity specific effects.

In summary, our findings indicate a significant role for the cerebellum in reactive inhibition, while there was no support for a role of the cerebellum in proactive inhibition. We acknowledge the speculative nature of the explanations, and further critical testing is warranted. Even though tDCS is effective in targeting the cerebellum, research on the mechanisms by which tDCS interacts with cerebellar tissue under different environmental conditions continues to be critical for understanding the neurological basis of behavior. Notwithstanding the lack of a mechanistic account, our results do provide direct evidence for a role of the cerebellum in reactive response inhibition, adding to the growing body of literature emphasizing the importance of the cerebellum in non-motor-related processes.

## References

[1] Buckner RL (2013). The cerebellum and cognitive function: 25 years of insight from anatomy and neuroimaging. Neuron..

[2] Leiner HC, Leiner AL, Dow RS (1993). Cognitive and language functions of the human cerebellum. Trends Neurosci.

[3] Oldrati V, Schutter DJLG (2018). Targeting the human cerebellum with transcranial direct current stimulation to modulate behavior: a meta-analysis. Cerebellum.

[4] Schmahmann JD, Sherman JC (1998). The cerebellar cognitive affective syndrome. Brain J Neurol.

[5] Stoodley CJ, Schmahmann JD (2010). Evidence for topographic organization in the cerebellum of motor control versus cognitive and affective processing. Cortex.

[6] Schmahmann JD. The cerebellum and cognition. Neuroscience letters. 2018. 10.1016/j.neulet.2018.07.005 29997061

[7] Stoodley CJ, Schmahmann JD (2018). Functional topography of the human cerebellum. Handb Clin Neurol.

[8] Mauss IB, Bunge SA, Gross JJ (2007). Automatic emotion regulation. Soc Personal Psychol Compass.

[9] Riva D, Giorgi C (2000). The cerebellum contributes to higher functions during development: evidence from a series of children surgically treated for posterior fossa tumours. Brain.

[10] Hernaez-Goni P, Tirapu-Ustarroz J, Iglesias-Fernandez L, Luna-Lario P (2010). The role of the cerebellum in the regulation of affection, emotion and behaviour. Rev Neurol.

[11] Adamaszek M, D’Agata F, Ferrucci R, Habas C, Keulen S, Kirkby K (2017). Consensus paper: cerebellum and emotion. Cerebellum.

[12] Strata P (2015). The emotional cerebellum. Cerebellum.

[13] Chen Y, Kumfor F, Landin-Romero R, Irish M, Hodges JR, Piguet O (2018). Cerebellar atrophy and its contribution to cognition in frontotemporal dementias. Ann Neurol.

[14] Bomyea J, Taylor CT, Spadoni AD, Simmons AN (2018). Neural mechanisms of interference control in working memory capacity. Hum Brain Mapp.

[15] Rubia K, Smith AB, Taylor E, Brammer M (2007). Linear age-correlated functional development of right inferior fronto-striato-cerebellar networks during response inhibition and anterior cingulate during error-related processes. Hum Brain Mapp.

[16] Zhang S, Tsai SJ, Hu S, Xu J, Chao HH, Calhoun VD, Li CSR (2015). Independent component analysis of functional networks for response inhibition: inter-subject variation in stop signal reaction time. Hum Brain Mapp.

[17] Han SD, Boyle PA, Yu L, Fleischman DA, Arfanakis K, Bennett DA (2013). Ventromedial PFC, parahippocampal, and cerebellar connectivity are associated with temporal discounting in old age. Exp Gerontol.

[18] Ortiz N, Parsons A, Whelan R, Brennan K, Agan ML, O’Connell R (2015). Decreased frontal, striatal and cerebellar activation in adults with ADHD during an adaptive delay discounting task. Acta Neurobiol Exp (Wars).

[19] Rubia K, Halari R, Christakou A, Taylor E (2009). Impulsiveness as a timing disturbance: neurocognitive abnormalities in attention-deficit hyperactivity disorder during temporal processes and normalization with methylphenidate. Philos Trans R Soc Lond Ser B Biol Sci.

[20] Tanaka SC, Doya K, Okada G, Ueda K, Okamoto Y, Yamawaki S (2004). Prediction of immediate and future rewards differentially recruits cortico-basal ganglia loops. Nat Neurosci.

[21] Ferrucci R, Cortese F, Priori A (2015). Cerebellar tDCS: how to do it. Cerebellum.

[22] van Dun K, Bodranghien FCAA, Marien P, Manto MU (2016). tDCS of the cerebellum: where do we stand in 2016? Technical issues and critical review of the literature. Front Hum Neurosci.

[23] Nitsche MA, Paulus W (2000). Excitability changes induced in the human motor cortex by weak transcranial direct current stimulation. J Physiol.

[24] Oosterholt BG, Linden D, Maes JH, Verbraak MJ, Kompier MA. Burned out cognition-cognitive functioning of burnout patients before and after a period with psychological treatment. 2012. 10.5271/sjweh.3256 22025205

[25] Costantini AF, Hoving KL (1973). The effectiveness of reward and punishment contingencies on response inhibition. J Exp Child Psychol.

[26] White MJ (1981). Response selection and visual search. B Psychonomic Soc.

[27] Finn PR, Mazas CA, Justus AN, Steinmetz J (2002). Early-onset alcoholism with conduct disorder: go/no go learning deficits, working memory capacity, and personality. Alcohol Clin Exp Res.

[28] Trommer BL, Hoeppner JAB, Lorber R, Armstrong KJ (1988). The Go—No-Go paradigm in attention deficit disorder. Ann Neurol.

[29] Du W, Green L, Myerson J (2002). Cross-cultural comparisons of discounting delayed and probabilistic rewards. Psychol Rec.

[30] Myerson J, Green L, Warusawitharana M (2001). Area under the curve as a measure of discounting. J Exp Anal Behav.

[31] Cho SS, Ko JH, Pellecchia G, Van Eimeren T, Cilia R, Strafella AP (2010). Continuous theta burst stimulation of right dorsolateral prefrontal cortex induces changes in impulsivity level. Brain Stimul.

[32] Figner B, Knoch D, Johnson EJ, Krosch AR, Lisanby SH, Fehr E, Weber EU (2010). Lateral prefrontal cortex and self-control in intertemporal choice. Nat Neurosci.

[33] Yamasaki T, Ogawa A, Osada T, Jimura K, Konishi S (2018). Within-subject correlation analysis to detect functional areas associated with response inhibition. Front Hum Neurosci.

[34] Chan C, Hounsgaard J, Nicholson C (1988). Effects of electric fields on transmembrane potential and excitability of turtle cerebellar Purkinje cells in vitro. J Physiol.

[35] Bikson M, Rahman A (2013). Origins of specificity during tDCS: anatomical, activity-selective, and input-bias mechanisms. Front Hum Neurosci.

[36] Stagg CJ, Nitsche MA (2011). Physiological basis of transcranial direct current stimulation. Neuroscientist.

[37] Jörntell H, Bengtsson F, Schonewille M, De Zeeuw CI (2010). Cerebellar molecular layer interneurons–computational properties and roles in learning. Trends Neurosci.

[38] VaezMousavi M, Barry RJ, Rushby J, Clarke A. Evidence for differentiation of arousal and activation in normal adults. 2007. 10.55782/ane-2007-1646 17691226

[39] Verbruggen F, Logan GD (2008). Automatic and controlled response inhibition: associative learning in the go/no-go and stop-signal paradigms. J Exp Psychol Gen.

[40] Ito M (1982). Cerebellar control of the vestibulo-ocular reflex--around the flocculus hypothesis. Annu Rev Neurosci.

[41] Ito M, Yamaguchi K, Nagao S, Yamazaki T (2014). Long-term depression as a model of cerebellar plasticity. Prog Brain Res.

[42] Brem A-K, Fried PJ, Horvath JC, Robertson EM, Pascual-Leone A (2014). Is neuroenhancement by noninvasive brain stimulation a net zero-sum proposition?. NeuroImage..

[43] Brunamonti E, Chiricozzi FR, Clausi S, Olivito G, Giusti MA, Molinari M, Ferraina S, Leggio M (2014). Cerebellar damage impairs executive control and monitoring of movement generation. PLoS One.

[44] de Water E, Cillessen AH, Scheres A (2014). Distinct age-related differences in temporal discounting and risk taking in adolescents and young adults. Child Dev.

[45] Ohmura Y, Takahashi T, Kitamura N (2005). Discounting delayed and probabilistic monetary gains and losses by smokers of cigarettes. Psychopharmacology..

[46] Scheres A, Dijkstra M, Ainslie E, Balkan J, Reynolds B, Sonuga-Barke E, Castellanos FX (2006). Temporal and probabilistic discounting of rewards in children and adolescents: effects of age and ADHD symptoms. Neuropsychologia..

[47] Murphy CM, Christakou A, Giampietro V, Brammer M, Daly EM, Ecker C, Johnston P, Spain D, Robertson DM, Murphy DG, Rubia K, MRC AIMS Consortium (2017). Abnormal functional activation and maturation of ventromedial prefrontal cortex and cerebellum during temporal discounting in autism spectrum disorder. Hum Brain Mapp.

[48] Zhang Y, Larcher KM-H, Misic B, Dagher A (2017). Anatomical and functional organization of the human substantia nigra and its connections. eLife..

[49] Schuller CB, Kuhn J, Jessen F, Hu XC (2019) Neuronal correlates of delay discounting in healthy subjects and its implication for addiction: an ALE meta-analysis study. Am J Drug Alcohol Ab 45(1):51–66. 10.1080/00952990.2018.1557675 30632802

